# Anti-diabetic effect of sorghum extract on hepatic gluconeogenesis of streptozotocin-induced diabetic rats

**DOI:** 10.1186/1743-7075-9-106

**Published:** 2012-11-27

**Authors:** Jungmin Kim, Yongsoon Park

**Affiliations:** 1 Department of Food and Nutrition, Hanyang University, 222 Wangsimni-ro, Seoul, Seongdong-gu, 133-791, Korea

**Keywords:** Diabetic rats, Hepatic gluconeogenetic enzyme expression, Sorghum

## Abstract

**Background:**

It has been suggested that Sorghum, a rich source of phytochemicals, has a hypoglycemic effect, but the mechanism is unknown. We investigated the effects of oral administration of sorghum extract (SE) on hepatic gluconeogenesis and the glucose uptake of muscle in streptozotocin-induced diabetic rats for six weeks.

**Methods:**

Male Wistar rats were divided in five groups (n=5 per group): normal control (NC), rats with STZ-induced diabetic mellitus (DM), diabetic rats administrated 0.4 g/kg body weight of SE (DM-SE 0.4) and 0.6 g/kg body weight of SE (DM-SE 0.6), and diabetic rats administrated 0.7 mg/kg body weight of glibenclamide (DM-G).

**Results:**

Administration of SE and G reduced the concentration of triglycerides, total and LDL-cholesterol and glucose, and the area under the curve of glucose during intraperitoneal glucose tolerance tests down to the levels observed in non-diabetic rats. In addition, administration of 0.4 and 0.6 g/kg SE and 0.7 mg/kg glibenclamide (G) significantly reduced the expression of phosphoenolpyruvate carboxykinase and the phosphor-p38/p38 ratio, while increased phosphor adenosine monophosphate activated protein kinase (AMPK)/AMPK ratio, but the glucose transporter 4 translocation and the phosphor-Akt/Akt ratio was significantly increased only by administration of G.

**Conclusions:**

These results indicate that the hypoglycemic effect of SE was related to hepatic gluconeogenesis but not the glucose uptake of skeletal muscle, and the effect was similar to that of anti-diabetic medication.

## Background

Diabetes mellitus is a metabolic disease characterized by chronic hyperglycemia [1] caused by increased hepatic glucose production [2] or abnormal glucose use in skeletal muscle [3]. Phosphoenolpyruvate carboxykinase (PEPCK), an important enzyme of gluconeogenesis, is regulated by the adenosine monophosphate activated protein kinase (AMPK) or p38 pathways [4,5]. Additionally, glucose transporter (GLUT4) is a rate-limiting factor for glucose uptake in skeletal muscle, and Akt (protein kinase B) is a central mediator of insulin-induced GLUT4 translocation from cytosol to membrane [6]. These protein expressions have been shown to be associated with the pathogenesis in streptozotocin (STZ)-induced diabetic rats [7-9].

Because of the adverse effects of diabetic medication such as the pain by injection, insulin resistance or hypoglycemic symptoms, there is increasing investigation into the use of herbs and plants for the treatment of diabetic mellitus. *Sorghum bicolor L. Monech* is the fifth most important cereal crop worldwide, both in terms of planted area and metric tons harvested [10]. Sorghum flour is a rich source of phytochemicals, tannins, phenolic acids, anthocyanins, phytosterols, and policosanols, and these bioactive components have been reported to have antioxidant [11,12], anti-carcinogenic [13], and cholesterol-lowering properties [14,15]. Previously, sorghum extracts have been shown to have hypoglycemic activity in STZ-induced diabetic rats [16]; however, the mechanism is unclear. Thus, we investigated the hypothesis that sorghum extract (SE) has hypoglycemic effects through inhibition of hepatic gluconeogenic enzymes and/or the increase in glucose uptake in skeletal muscle in STZ-induced diabetic rats.

## Methods

### Animals and diet

Protocol approved by the Institutional Animal Care and Use Committee of Hanyang University was used for all animal experiments (HY-IACUC-11-059). Six week old male Wistar rats (Orient, Gyeonggi-do, Korea) were housed in individual ventilated in an air-conditioned room maintained at 22±2°C with a 12 h light–dark cycle. After one week of acclimatization, twenty rats were intraperitonially injected with STZ (70 mg/kg body weight in 0.05 M citrate buffer; Sigma Co, St. Louis, MO, USA) once, and then blood glucose concentration was measured to confirm the development of diabetes mellitus. After 3 days of STZ injection, twenty STZ-induced diabetic rats and five normal rats without STX injection were fed with an AIN-93M diet (Research Diets, New Brunswick, NJ, USA) and water *ad libitum* for 6 weeks, and the body weight and food intake were recorded every week.

Sorghum (Sorghum bicolor L. Moench cv. Hwanggeumchal-susu) was grown at the Department of Functional Crops, National Institute of Crop Science, Rural Development Administration, Milyang, Korea during the 2010 growing season. Voucher herbarium specimens were deposited with the reference number (KNICS-579) in the Herbarium of the Department of Functional Crops. The botanical identification was made by Dr. Ill-Min Chung of Konkuk University [16]. Sorghum was finely ground using a Pin-type Mill (DK-201, Sejung Tech, Daegu, Korea) and extracted using 80% fermented ethanol at room temperature for 24 h while shaking (WiseCube WIS-RL010, Daihan Scientific Co., Ltd., Seoul, Korea). The extracts were centrifuged at 5,000 g for 30 min and the supernatants were filtered through Advantec 2 filter paper (Advantec Toyo Kaisha Ltd., Tokyo, Japan). The filtrates were evaporated using a rotary evaporator (Eyela N-1000, Tokyo Rikakikai Co., Tokyo, Japan) at 40°C, and then freeze-dried in vacuum (FDT-8612, OPERON, Kimpo, Korea). The dried extract was stored at −20°C.

During a six-week period of diet, normal control rats (NC) were orally administrated 0.1 mL of saline, and twenty STZ-induced diabetic rats were randomly divided into four groups that orally administrated either 0.1 mL of saline (DM) or 0.1 mL of saline with a 0.4 g/kg body weight of SE (DM-SE 0.4), 0.6 g/kg body weight of SE (DM-SE 0.6) or 0.7 mg/ kg body weight of glibenclamide (DM-G) using gavage (n = 5 per group).

Intraperitoneal glucose tolerance tests (IPGTT) were performed by intraperitoneal injection of 25% glucose (2 g/kg body weight) on the last day of the experiment after an overnight fast. Blood samples were collected from the tail vein at 30, 60, and 120 min after injection.

At the end of the experimental period, all rats were anesthetized with an intraperitoneal injection of tiletamine (25 mg/kg), zolazepam (25 mg/kg), and xylazine (10 mg/kg) after overnight fasting. Blood was collected into SST tubes (BD Vacutainer, Franklin Lakes, NJ, USA) and centrifuged at 3000 g for 15 min (HA 1000–3, Hanil Sciences Industrial CO. Ltd., Incheon, Korea). Organs and adipose tissues were harvested, rinsed with saline and then weighed [17]. Serum and tissue samples were stored at −80°C.

### Biochemical assays

Glucose concentration during IPGTT was determined with AccuCheck (Roche Diagnostics, Indianapolis, IN, USA). The values of the area under the glucose time curve (AUC) were calculated using the glucose levels at each time point during IPGTT. The serum levels of triglycerides, total- and HDL-cholesterol, glucose, glutamic oxaloacetic transaminase (GOT), and glutamic pyruvic transaminase (GPT) were determined using a commercially available kit (Asan Pharm., Hwaseong, Korea) with a spectrophotometer (DU 600, Beckman Coulter, Inc., Indianapolis, IN, USA). LDL-cholesterol concentration was calculated using the Friedwald formula. The serum insulin level was determined using an ultra-sensitive rat insulin enzyme linked immunosorbent assay kit (Crystal Chem, Downers Grove, IL, USA) with a microplate reader (iMark, Bio-Rad Laboratories, Hercules, CA, USA).

### Western blotting

Liver and skeletal muscles were homogenized in a 0.8 ml ice-cold lysis buffer (20 mM HEPES, 0.25 M sucrose, 0.5 mM EDTA, 2 mM dithiothreitol, 1 mM PMSF, 10 μg/mL leupeptin, 10 μg/mL aprotinin, and 1mM Na_3_VO_4_, pH 7.5). The homogenates were centrifuged at 10,000 g for 15 min at 4°C, and the supernatant was centrifuged at 20,000 g for 1 h at 4°C to obtain cytosolic fraction. For the membrane fraction of skeletal muscle, the pellets were re-suspended in 250 μl of lysis buffer and 1% (v/v) triton X-100, incubated on ice for 30 min, and centrifuged at 200,000 g for 30 min at 4°C. The protein concentrations were determined using a Bradford assay with bovine serum albumin (Bio-Rad, Hercules, CA, USA) as the standard. Equal amounts of protein (30μg) from liver or skeletal muscle was separated on 8% SDS-PAGE and transferred to a polyvinylidine fluoride membrane (0.45 μm, Immobilon-P transfer membrane, Millipore, USA). After blocking, the membranes were incubated overnight with a primary antibody for PEPCK (1:1,000, Cell Signaling Technology, Beverly, MA, USA), p38 (1:1,000, Cell Signaling Technology, Beverly, MA, USA), phospho p-38 (1:500, Cell Signaling Technology, Beverly, MA, USA), AMPK (1:1,000, Cell Signaling Technology, Beverly, MA, USA), phospho AMPK (1:500, Cell Signaling Technology, Beverly, MA, USA), GLUT4 (1:2000, abCam, Cambridge, UK), Akt (1:1,000, Cell Signaling Technology, Beverly, MA, USA), or phospho-Akt (1:500, Cell Signaling Technology, Beverly, MA, USA) in Tris-buffered saline with Tween 20 (TBST) containing 5% nonfat milk at 4°C. After washing in TBST, the membranes were incubated with horseradish peroxidase conjugated goat anti-rabbit or mouse IgG (1:5,000, Cell Signaling Technology, Beverly, MA, USA) for 1 h. The immunoreactive signals were developed using an enhanced chemiluminescence kit (GE Healthcare Life Sciences, Piscataway, NJ, USA) and exposed to Kodak film. The relative and normalized protein expression was calculated by β-action (1:1,000, BD Transduction Laboratories, NJ, USA).

### Statistical analysis

All data are expressed as the mean ± standard error of the mean (SEM). Statistical differences among the groups were calculated by the analysis of variance (ANOVA) followed by Duncan’s multiple range test (SPSS 18.0 version., Chicago, IL, USA). Differences with p < 0.05 were considered significant.

## Results

### Food intake, body weight, and organ weights

Dietary intake, and weight of liver and kidney as percentage of body weight were significantly greater in all DM groups than in the NC group, but body weight and weight of retroperitoneal, epididymal and perirenal adipose tissues were significantly lower in all DM groups than in the NC group (Table 1). Among the DM groups, there were no significant differences in dietary intake, body weight and weight of adipose tissue, liver and kidney.

**Table 1:** Dietary intake, body weight and various organ weights

	**NC**	**DM**	**DM-SE 0.4**	**DM-SE 0.6**	**DM-G**
Dietary intake (g/day)	22.10±0.23^a^	25.94±1.30^b^	26.65±1.60^b^	28.41±1.26^b^	27.11±1.27^b^
Initial body weight (g/day)	246.20±6.76^a^	208.60±7.79^b^	213.20±7.36^b^	206.60±9.27^b^	207.80±14.15^b^
Final body weight (g/day)	491.40±9.01^a^	375.40±43.17^b^	347.60±35.94^b^	385.40±30.44^b^	323.40±38.45^b^
Liver (mg/g body weight)	11.52±0.57^a^	11.40±0.66^b^	12.27±0.63^b^	12.04±0.30^b^	10.83±0.69^b^
kidney (mg/g body weight)	2.73±0.10^a^	2.91±0.07^b^	2.65±0.08^b^	2.72±0.06^b^	2.83±0.11^b^
Retroperitoneal fat (g)	12.11±1.07^a^	8.52±0.62^b^	8.32±1.60^b^	8.99±0.56^b^	6.78±0.34^b^
Epididymal fat (g)	11.82±0.81^a^	8.97±0.27^b^	6.73±1.44^b^	8.68±0.35^b^	6.40±1.27^b^
Perirenal fat (g)	0.99±0.21	0.93±0.15	0.94±0.15	0.75±0.15	0.84±016

Values are expressed as the mean ± SEM (n = 5); NC, normal control rats administrated saline; DM, rats with diabetes mellitus administrated saline; DM-SE 0.4, rats with diabetes mellitus administrated 0.4 g/kg body weight of sorghum extract; DM-SE 0.6, rats with diabetes mellitus administrated 0.6 g/kg body weight of sorghum extract; DM-G, rats with diabetes mellitus administrated 0.7 mg/kg body weight of glibenclamide. The values in the rows with different letters are significantly different at *p* < 0.05 using ANOVA with Duncan’s multiple range test.

### Levels of glucose, insulin lipid profiles, and liver function

**Figure 1:**
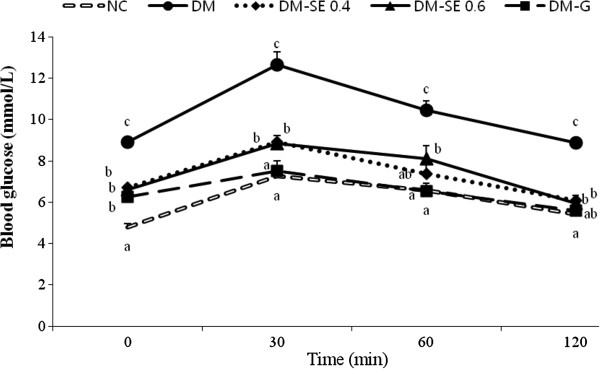
**Blood glucose levels during the intraperitoneal glucose tolerance tests.** NC, normal control rats administrated saline; DM, rats with diabetes mellitus administrated saline; DM-SE 0.4, rats with diabetes mellitus administrated 0.4 g/kg body weight of sorghum extract; DM-SE 0.6, rats with diabetes mellitus administrated 0.6 g/kg body weight of sorghum extract; DM-G, rats with diabetes mellitus administrated 0.7 mg/kg body weight of glibenclamide. The values are mean ± SEM (n = 5). Values with different superscripts are significantly different at p < 0.05 using ANOVA with Duncan’s multiple range test.

Serum concentrations of triglycerides, and total- and LDL-cholesterol were significantly lower in the DM-SE 0.4 , DM-SE 0.6, DM-G, and NC groups than the DM group, while HDL-cholesterol level was significantly lower in DM-G than the other DM and NC groups (Table 2). There were no significant differences in serum levels of glutamic oxaloacetic transaminase and glutamic pyruvic transaminase, suggesting that SE had no harmful effect on liver function. Serum glucose concentration and AUC during IPGTT were significantly lower in DM-SE 0.6 and DM-G than in DM, but not DM-SE 0.4 (Table 2). Serum insulin level in DM-G was significantly increased up to that of NC, but SE had no significant effect on insulin concentration. Blood glucose levels reached a peak at 30 min during IPGTT (Figure 1), and the peak concentrations of glucose were significantly lower in DM-SE 0.4 and DM-SE 0.6 than DM, but higher than NC and DM-G (Figure 1).

**Table 2:** Lipid profiles, liver function, and levels of glucose and insulin in serum

	**NC**	**DM**	**DM-SE 0.4**	**DM-SE 0.6**	**DM-G**
Triglycerides (mmol/L)	0.92±0.15^a^	1.86±0.13^b^	0.75±0.13^a^	0.68±0.12^a^	0.89±0.02^a^
Total cholesterol (mmol/L)	2.01±0.10^a^	2.70±0.19^b^	1.81±0.05^a^	1.81±0.06^a^	2.14±0.10^a^
HDL-cholesterol (mmol/L)	1.20±0.04^a^	1.33±0.02^a^	1.28±0.02^a^	1.34±0.06^a^	1.02±0.09^b^
LDL-cholesterol (mmol/L)	0.73±0.03^a^	1.25±0.07^b^	0.82±0.07^a^	0.72±0.04^a^	0.88±0.05^a^
Glutamic oxaloacetic transaminase (IU/L)	42.81±1.19	46.02±2.92	42.94±2.05	44.26±0.73	45.03±1.52
Glutamic pyruvic transaminase (IU/L)	14.54±3.45	12.72±1.20	13.78±1.94	10.17±0.73	9.68±1.96
Glucose (mmol/L)	1.18±0.06^a^	1.67±0.12^c^	1.51±0.07^bc^	1.29±0.02^b^	1.21±0.12^a^
Insulin (pmol/L)	3.73±0.27^a^	1.25±0.20^b^	1.64±0.26^ab^	1.91±0.11^ab^	3.69±1.46^a^
AUC of glucose (mmol·min/L)	748.25±27.39^a^	1250.25±35.24^c^	881.20±39.61^b^	907.76±29.55^b^	781.39±23.36^a^

Values are expressed as mean ± SEM (n = 5); NC, normal control rats administrated saline; DM, rats with diabetes mellitus administrated saline; DM-SE 0.4, rats with diabetes mellitus administrated 0.4 g/kg body weight of sorghum extract; DM-SE 0.6, rats with diabetes mellitus administrated 0.6 g/kg body weight of sorghum extract; DM-G, rats with diabetes mellitus administrated 0.7 mg/kg body weight of glibenclamide; AUC, area under the curve during intraperitoneal glucose tolerance tests. The values in the rows with different letters were significantly different at p < 0.05 using ANOVA with Duncan’s multiple range test.

### Protein expression in liver and muscle

**Figure 2:**
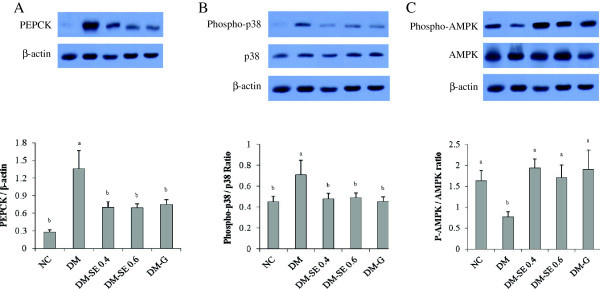
**Expression of phosphoenolpyruvate carboxykinase (PEPCK), adenosine monophosphate activated protein kinase (AMPK), and p38.** NC, normal control rats administrated saline; DM, rats with diabetes mellitus administrated saline; DM-SE 0.4, rats with diabetes mellitus administrated 0.4 g/kg body weight of sorghum extract; DM-SE 0.6, rats with diabetes mellitus administrated 0.6 g/kg body weight of sorghum extract; DM-G, rats with diabetes mellitus administrated 0.7 mg/kg body weight of glibenclamide. The values are mean ± SEM (n = 5). The values with different superscripts are significantly different at p < 0.05 using ANOVA with Duncan’s multiple range test.

**Figure 3:**
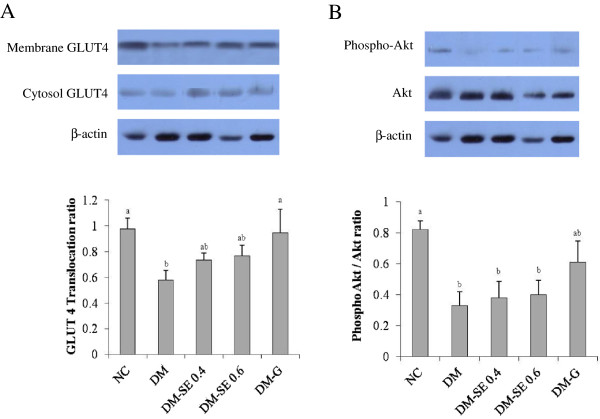
**Expression of the glucose transporter (GLUT) 4 and Akt (protein kinase B).** NC, normal control rats administrated saline; DM, rats with diabetes mellitus administrated saline; DM-SE 0.4, rats with diabetes mellitus administrated 0.4 g/kg body weight of sorghum extract; DM-SE 0.6, rats with diabetes mellitus administrated 0.6 g/kg body weight of sorghum extract; DM-G, rats with diabetes mellitus administrated 0.7 mg/kg body weight of glibenclamide. The values are mean ± SEM (n = 5). The values with different superscripts are significantly different at p < 0.05 using ANOVA with Duncan’s multiple range test.

The expression of PEPCK and the phosphor-p38/p38 ratio were significantly lower, while the phospho-AMPK/AMPK ratio was significantly higher in NC, DM-SE 0.4, DM-SE 0.6, and DM-G than in DM, suggesting that SE and G decreased hepatic gluconeogenesis (Figure 2). Phosphorylation of Akt and GLUT4 translocation was significantly decreased in DM as compared with NC, but SE had no significant effect (Figure 3). On the other hand, GLUT4 translocation was significantly increased by the administration of G, suggesting that G reduced blood glucose levels by both reducing hepatic gluconeogenesis and increasing glucose uptake by muscle.

## Discussion

The present study demonstrates that oral administration of SE significantly reduces blood glucose concentration in STZ-induced diabetic rats by inhibiting hepatic gluconeogenesis through suppression of PEPCK and p38 expression and increases AMPK expression. However, SE had no significant effect on glucose uptake by skeletal muscle determined by GLUT4 translocation and Akt phosphorylation, suggesting that the hypoglycemic effects of SE may be related to an insulin independent pathway.

These hypoglycemic effects were consistent with the results of a recent study that found that SE rich tannins at dosages of 0.25-0.5 g/kg of body weight significantly decreased serum glucose concentration in STZ-induced diabetic rats [16] and mice fed a high fat diet [18]. A previous study showed that SE strongly inhibited *in vitro* activities of α-glucosidase and α-amylase, targets for the development of diabetic drugs [19]. Lakshmi et al. [20] also reported that sorghum grain significantly reduced the fasting glucose level and the AUC of glucose in type 2 diabetic patients. The SE used in the present study was 0.4-0.6 g/kg of body weight, which was translated as 20–30 g of sorghum grain per kg of body weight for human. Due to the low yield rate, it may not be achievable to reduce blood glucose levels by consumption of intact sorghum grain.

In the present study, administration of SE significantly reduced the expression of PEPCK and phosphor-p38, while increasing phosphor-AMPK. Expression of PEPCK is an important factor responsible for hepatic gluconeogenesis [2,21], and has previously been shown to increase in the liver of diabetic rats [7,8]. In addition, the p38 pathway increased the expression of PEPCK [8,22,23], but the p38-activated PEPCK signal pathway has been shown to be inhibited by AMPK [8,24]. It has also been reported that the AMPK-α 2 catalytic subunit is a key target for the regulation of hepatic glucose production by adiponectin and leptin but not insulin, suggesting that AMPK is regulated by a mechanism distinct from insulin [7,25,26]. Since we observed that SE significantly reduced blood glucose concentration but did not significantly change insulin levels, these results indicate that SE reduces hepatic gluconeogenesis through an insulin independent pathway.

GLUT4 is the rate-limiting step for glucose uptake by an insulin dependent pathway in skeletal muscle [27], and GLUT4 translocation from cytosol to the plasma membrane is regulated by Akt [9,28]. Previous studies have observed that blood glucose levels increase with reductions of insulin concentration in GLUT4 knockout mice [29]. Additionally, Akt and GLUT4 translocation of skeletal muscle was reduced in STZ-induced diabetic rats [8] and impaired in diabetic patients [30]. Zhou et al. [26] consistently reported that glucose uptake was not induced by phosphorylation of Akt nor the expression and translocation of GLUT4 when insulin levels were low. In the present study, administration of SE had no significant effect on GLUT 4 translocation and the phosphor-Akt/Akt ratio, suggesting that the hypoglycemic effect of SE was not related to glucose uptake by skeletal muscle.

In this study, administration of SE also significantly decreased the concentration of triglycerides as well as total- and LDL-cholesterol. Previous studies have consistently showed that SE decreases cholesterol levels by reducing hepatic cholesterol synthesis and increasing cholesterol excretion into feces [6,11]. Chung et al. [16] also observed that SE markedly decreased the serum concentration of total-cholesterol, suggesting that HMG-CoA reductase might be responsible. Therefore, these results indicate that SE may have beneficial bioactive components that could exert blood lipid profiles in humans [11], but whether SE has the ability to reduce cholesterol synthesis through HMG-CoA reductase in vivo warrants further investigation.

Our study had a few limitations. We did not measure the active components in the 80% ethanol extract of sorghum or determine whether the protein expression was due to a change in mRNA levels. We also did not observe the dose dependency of SE. In conclusion, the oral administration of SE significantly reduced blood glucose concentration by inhibition of hepatic gluconeogenesis, particularly via suppression of PEPCK and p38 expression, and increased AMPK expression in STZ-induced diabetic rats.

## Abbreviations

Akt: Protein kinase B; AMPK: Adenosine monophosphate activated protein kinase; G: Glibenclamide; GLUT: Glucose transporter; p38: p38 mitogen-activated protein kinase; PEPCK: Phosphoenolpyruvate carboxykinase; SE: Sorghum extract; STZ: Streptozotocin.

## Competing interests

The authors declare that they have no competing interest.

## Authors’ contributions

JK conducted the research and wrote the manuscript. YP designed the research and had primary responsibility for the final content. All authors read and approved the final manuscript.
